# α-Ketoglutaramate—A key metabolite contributing to glutamine addiction in cancer cells

**DOI:** 10.3389/fmed.2022.1035335

**Published:** 2022-11-04

**Authors:** Arthur J. L. Cooper, Thambi Dorai, John T. Pinto, Travis T. Denton

**Affiliations:** ^1^Department of Biochemistry and Molecular Biology, New York Medical College, Valhalla, NY, United States; ^2^Department of Urology, New York Medical College, Valhalla, NY, United States; ^3^Department Pharmaceutical Sciences, College of Pharmacy and Pharmaceutical Sciences, Washington State University Health Sciences Spokane, Spokane, WA, United States; ^4^Department of Translational Medicine and Physiology, Elson S. Floyd College of Medicine, Washington State University Health Sciences Spokane, Spokane, WA, United States; ^5^Steve Gleason Institute for Neuroscience, Washington State University Health Sciences Spokane, Spokane, WA, United States

**Keywords:** ω-amidase, glutaminase II pathway, glutamine transaminase K, glutamine transaminases, α-ketoglutaramate, α-ketoglutarate, methionine salvage pathway, nitrogen homeostasis

## Introduction

**FIGURE 1:**
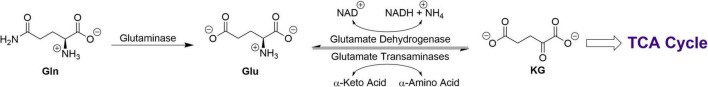
The glutaminase I pathway for the production of KG from Gln.

Many rapidly dividing cells, including various cancers, rely on L-glutamine (Gln) as a major energy/carbon/nitrogen source. For cancers this process is named “glutamine addiction” [e.g., (1–11)]. Almost all researchers studying glutamine addiction assume that only one major pathway contributes to this phenomenon. In this pathway, which is referred to here as the glutaminase I pathway (Figure 1), Gln is converted to L-glutamate (Glu) and ammonia by the action of a glutaminase, followed by conversion of Glu to α-ketoglutarate (KG) by a transaminase (aminotransferase) or by glutamate dehydrogenase (GDH). The KG thus formed can then enter the tricarboxylic acid (TCA) cycle. However, as outlined below, normal and cancerous tissues can utilize an alternative pathway (the glutaminase II pathway) for the formation of KG from Gln.

## Glutaminase I and glutaminase II [glutamine transaminase—ω-amidase (GTωA)] pathways

Rat liver glutaminase (Figure 1) was discovered in the 1940s and designated glutaminase I or (since it is activated by phosphate) phosphate-activated glutaminase (PAG) (12, 13). Subsequently, it was discovered that mammalian tissues contain two PAG isozymes, namely, a kidney type (KGA, GLS, GLS1) and its active splice variant (GAC) and a liver type (LGA, GLS2) and its active splice variant (GAB) (14–16).

**FIGURE 2:**
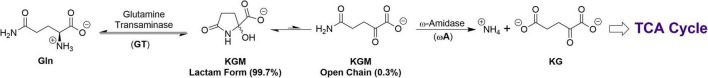
The GTωA pathway for production of KG from Gln. Note the relatively low concentration of the open-chain (substrate) form of KGM.

Subsequently, Meister et al. showed that rat liver possesses a Gln transaminase (GT) that catalyzes the transamination of Gln to α–ketoglutaramate (KGM) in the presence of an α-keto acid acceptor, which is coupled to an enzyme (ω–amidase; ωA) that catalyzes the hydrolysis of KGM to KG (Figure 2) (17, 18). This pathway was named the glutaminase II pathway and we have retained this nomenclature in our previous publications [e.g., (19, 20)]. Unfortunately, researchers have overlooked this pathway, possibly because of confusion in nomenclature between the glutaminase II pathway and glutaminase 2 isozyme. To obviate this problem, we now refer to the glutaminase II pathway as the glutamine transaminase—ω-amidase (GTωA) pathway. *We propose here that KGM—a key intermediate of this pathway—facilitates glutamine addiction in cancer cells*.

## Metabolic importance of the glutamine transaminase—ω-amidase pathway

We have suggested that the GTωA pathway salvages α-keto acids arising through non-specific transamination of L-amino acids by converting them back to the original L-amino acid (19, 20). Indeed, recent work supports this idea (21). We have also suggested that the GTωA pathway may be involved in cellular/intracellular transport of α-keto/L-amino acids (19, 20). Finally, we have emphasized the importance of the GTωA pathway in closing the methionine salvage pathway (MSP) (19, 20). During polyamine synthesis, carbon 1 (C_1_) of methionine is converted to CO_2_, C_2_-C_4_ are incorporated into polyamines and the sulfur and methyl are incorporated into 5′-methylthioadenosine (MTA). In the MSP, this sulfur and methyl group are incorporated into α-keto-γ-methiolbutyrate (KMB). The last step of the MSP is transamination of KMB to L-methionine. An important amine donor in this process is Gln (19, 20 and references quoted therein). Indeed, a homolog of mammalian glutamine transaminase K (GTK) closes the MSP in bacteria and plants (22). Moreover, KMB is an excellent substrate of two mammalian Gln transaminases, namely GTK and L (GTL) (19, 20).

**FIGURE 3:**
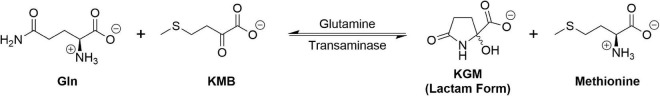
Closure of the MSP by transamination of KMB with Gln. For simplicity, the open-chain form of KGM is omitted since it represents only 0.3% of total KGM (see Figure 2).

KG can be generated through the GTωA pathway under anoxic conditions, provided α-keto acids are available for transamination with Gln. *This is a major advantage to dividing cancer cells, where the availability of oxygen may be limited.* But, there is also another advantage. Polyamines are important for both normal and neoplastic cell function and replication (e.g., 23). Closure of the MSP is depicted in Figure 3. *Thus, the GT*ω*A pathway is important in cancer both for closing the MSP* (Figure 3) *and for providing anaplerotic KG, even under anoxic conditions* (Figure 2).

## Glutamine transaminases

Meister et al. partially purified a Gln transaminase from rat liver and showed that it possessed wide α-keto acid substrate specificity (17, 18). Subsequently, Cooper and Meister showed that rat tissues contain two Gln transaminases, namely a liver type (GTL) and a kidney type (GTK) (24, 25). Both isozymes were shown to exhibit broad α-keto/L-amino acid substrate specificity (24, 25). This has led to some redundant/confusing nomenclature in the scientific literature. For example, GTK and GTL are identical to enzymes other researchers have named kynurenine aminotransferase 1 (KAT1/KYAT1) and kynurenine aminotransferase 3 (KAT3/KYAT3), respectively [see the discussion in 19, 20]. However, Li et al. have shown that, of all the L-amino acids tested (including L-kynurenine), the highest catalytic efficiency for human GTK/KAT1 and mouse GTL/KAT3 is with Gln (26, 27). Moreover, the average concentration of Gln in human tissues is ∼9 mM [calculated from data in Cruzat et al. (28)], whereas that of L-kynurenine is ∼22 μM in rat liver, with lower concentrations in brain, lung and spleen (29). Human KAT2 (KYAT2, α-aminoadipate aminotransferase) exhibits some activity toward Gln (30). Moreover, Gln transaminase and ω-amidase activities are present in every rat tissue examined (31, 32). *Taken together, the findings indicate that Gln transamination in mammalian tissues is extensive*.

## The glutamine transaminase—ω-amidase pathway in human tissues

The GTωA pathway was shown to be present in *freshly isolated human kidney tissue* (33–35). Moreover, Darmaun et al. investigated the turnover kinetics of [^15^N]Glu, [2-^15^N]Gln and [5-^15^N]Gln administered to adult human male volunteers and concluded that Gln transamination in humans may be extensive (36).

Both GTK/KAT1 and GTL/KAT3 possess mitochondrial leader sequences (37, 38)—alternative splicing results in activity in both cytosolic and mitochondrial compartments. ω-Amidase (annotated as Nit2 in the human genome) RNA has been detected in all sixteen human tissues investigated, with highest levels in liver and kidney (39). ω-Amidase activity has also been detected in cytosolic and mitochondrial fractions of rat tissues (19, 20 and references cited therein). The presence of a mitochondrial GTωA pathway is predicted to be advantageous to human cells, especially to rapidly dividing cancer cells. *Thus, as noted above, anaplerotic KG can be generated from Gln under anaerobic conditions as long as the tumor is supplied with* α*-keto acid substrates of the Gln transaminases*.

## Properties of α-ketoglutaramate

Meister presented evidence that open-chain KGM, in solution, is in equilibrium with a cyclic (lactam) form (2-hydroxy-5-oxoproline, Figure 2) (40). This observation was verified by Hersh who showed that, at neutral pH, the equilibrium favors the lactam (99.7%) over the open-chain form (0.3%) (41) (Figure 2). In fact, in the NMR spectrum of pure KGM, the open chain form (the actual substrate of ω-amidase) is undetectable (42). (Nevertheless, unless otherwise stated, the designation KGM herein is the sum total of open-chain and lactam forms).

A major reason why the GTωA pathway has been little studied is possibly because KGM is not available commercially and, until recently, could only be synthesized enzymatically. This procedure involves oxidation of Gln (solution neutralized with NaOH) by snake venom L-amino acid oxidase in the presence of oxygen and catalase (40). The generated KGM is separated from NH_4_^+^, Na^+^ and unreacted Gln on a Dowex 50[H^+^] column. The KGM eluted with water can be lyophilized to the free acid or converted to sodium or barium salts (40). This procedure has been repeated in our laboratory (43) and by others (44). However, Gln, at neutral pH, slowly cyclizes to 5-oxoproline (5-OP), with the elimination of ammonia (45), and this occurs during the enzymatic reaction to contaminate the final KGM product. Similarly, hydrolysis of KGM to KG occurs upon prolonged incubation. Thus, the enzymatic reaction must be carried out as quickly as possible. Nevertheless, KGM, prepared by the enzymatic procedure, invariably contains ∼1 to a few percent 5-OP (43, 44) and traces of KG.

In order to overcome problems associated with enzymatic synthesis and to scale-up production, a group led by one of us (TTD) recently published a procedure for the organic synthesis of KGM that is not contaminated with either KG or 5-OP (42). After this article was published, an article came to our attention in which the authors oxidized Gln at neutral pH with ammonium vanadate (NH_4_VO_3_) to a complex isolated in 65% yield as [V_2_O_3_ (2-hydroxy-5-oxoproline)_2_(2,2′-bipyridine)_2_]-7H_2_O (46). The possibility that cleanup of this complex will provide an alternative, convenient synthetic route to KGM lactam is currently being considered. *We anticipate that a convenient source of KGM will stimulate interest in the GT*ω*A pathway and especially the role of* ω*-amidase and its substrate KGM in cancer biology.*

## Clinical biochemistry of α-ketoglutaramate—relevance to cancer?

Rat liver ω-amidase has a broad pH optimum (∼5.0–9.5), with α-ketosuccinamate (KSM, the α-keto acid analog of asparagine) as the substrate, yet the pH optimum with the next higher homolog of KSM (i.e., KGM) is much narrower, with an apparent pH optimum of ∼9.0 and very little activity at pH values below 7.5 (40). Hersh showed that the rate of interconversion between lactam and open-chain forms of KGM is base (OH^–^)-catalyzed and, thus, favors faster rate of open-chain formation of KGM at high pH values (41). At pH values ≥ 8.5, the rate of interconversion is so rapid that it is unlikely to be rate-limiting for enzyme activity. Thus, ω-amidase activity measurements with KGM as substrate are usually conducted at pH values of 8.5–9.0. Since KSM does not cyclize, there is no pH limitation to enzyme activity over the pH range of 5.0–9.5.

Due to the very low relative concentration of open-chain KGM compared to its lactam, at pH 7.0–7.2, this compound is expected to accumulate to measurable steady state concentrations in mammalian tissues in this pH range, even though ω-amidase activity is inherently high. Indeed, normal concentrations of KGM in several rat tissues are 6 μM to 216 μM (47, 48).

KGM was shown to accumulate in the cerebrospinal fluid (CSF) of hyperammonemic patients with hepatic encephalopathy (HE) (49, 50). KGM also accumulates in the urine of hyperammonemic patients with (1) disorders of the urea cycle (51, 52) and (2) citrin deficiency (53). Evidently, KGM is a biomarker for hyperammonemia associated with several disorders. The increase in KGM may be related to the hyperammonemia-induced increase in Gln concentrations, leading to increased Gln transamination (49, 52).

Many cancer cells convert glucose to lactate even in the presence of adequate oxygen (Warburg effect). This phenomenon is associated with the acidification of the microenvironment (54). However, recent evidence suggests a reverse pH gradient in tumors (55). The pH optimum of GTK and GTL is ∼8.0 to 9.0 (26, 27). Thus, an alkaline environment will favor Gln transamination, whereas an acidic environment will have the reverse effect. Additionally, it is predicted that lower pH values will favor higher steady state concentrations of KGM. Thus, concentrations of KGM throughout the tumor will be affected, in part, by Gln transaminase activity, ω-amidase activity and pH gradients. Moreover, ammonia generated from Gln is a tumor promoter (56). However, the relationship between ammonia and KGM concentrations is uncertain. Whether KGM is a cancer biomarker remains to be established.

Interestingly, in a study from Johns Hopkins University (JHU) it was reported that KGM could be detected by ^1^H NMR in patient-derived pancreatic cancer (JHU094) orthotopic tumors in nude mice (57). Moreover, the KGM tumor concentration increased in mice in which GLS1 was inhibited, most likely as a result of increased Gln transamination in the face of inhibited GLS1 activity (57). This suggests a metabolic shift in cancer cells from reliance on the glutaminase I pathway to the GTωA pathway. The JHU group also showed that the median survival time was significantly increased in nude mice carrying a human pancreatic cancer xenograft in which GTK was knocked down, compared to controls carrying xenografts expressing normal GTK activity (57).

## The glutamine transaminase—ω-amidase pathway and glutaminase 1 in cancerous tissues

Thul et al. demonstrated that GTK (annotated as KYAT1/CCBL1), GTL (annotated as KYAT3/CCBL2) and ω-amidase (annotated as Nit2) occur in several cancer cell lines, including A-431, U-251MG and U2OS (58). The human protein atlas^1^ also lists the presence of the protein and mRNA for GTK, GTL and ω-amidase in many human cancers. Below, we provide additional information on the GTωA pathway in cancers.

Medulloblastoma is the most common malignant cancer in children and is associated with a poor prognosis [(59) and references cited therein]. Pham et al. investigated the isotopomer patterns in L-glutamate generated from L-[U-^13^C]Gln and deduced that the D425MED orthotopic human *Myc*-amplified medulloblastoma tumors in nude mice preferentially utilize the GTωA pathway to maintain glutamine addiction (59). Moreover, *KYAT* (i.e., the gene for GTK/KAT1), and its mRNA, are upregulated in medulloblastoma, compared to other pediatric brain tumors, in the Children’s Brain Tumor Network/KidsFirst Pediatric Brain Tumor Atlas RNAseq dataset (59).

Pan et al. (60) and Zhang et al. (61) reported an increase in GLS1 expression with increasing aggressiveness of human prostate cancer cells. We also found this to be the case (62). This is consistent with GLS1 playing a prominent role in the anaplerotic supply of KG in human prostate cells. We also showed the importance of the GTωA pathway in this process in several human cancers. Thus, immunohistochemistry studies from our laboratory showed that GTK/ω-amidase are well represented in human bladder, pancreatic and prostate cancers (19, 20, 62, 63). An especially intriguing finding is the discovery that specific activities of glutamine transaminase(s) and ω-amidase are relatively high in rat prostate (62). Moreover, as was found to be the case with GLS1, we showed that the amount of GTK and ω-amidase protein in prostate cancer cells, in culture, increases with increasing aggressiveness of the cells (62). These findings are consistent with the ability of the prostate to produce polyamines and large amounts of citrate that are excreted into the seminal fluid (references cited in 62). The coupling of the MSP to the GTωA pathway (Figure 3) not only allows methionine to be regenerated following polyamine biosynthesis, but also allows for the anaplerotic replenishment of citrate carbon lost in the semen. Thus, both GLS1 and enzymes of the GTωA pathway are upregulated and adaptive to tumor cell environment in human prostate cancer and can contribute to KG anaplerosis.

## Conclusion—Would an ω-amidase inhibitor be useful in cancer therapy?

Several researchers have suggested that glutaminase inhibitors [e.g., BPTES (bis-2-(5-phenylacetamido-1,3,4-thiadiazol-2-yl)ethyl sulfide] or analogs] alone, or in combination with other drugs, may be useful anti-cancer agents [e.g., (64–66)]. Unfortunately, blocking Gln anaplerosis by utilizing the highly potent allosteric GLS inhibitor CB-839 as a monotherapeutic intervention, has not been clinically successful. Nevertheless, there are numerous, active ongoing clinical trials utilizing a combination of CB-839 with FDA approved chemotherapies (e.g., www.clinicaltrials.gov identifiers NCT02861300, NCT03798678). *We suggest that, once GLS1 is inhibited, the cancer cells will begin to rely more heavily on the GT*ω*A pathway for the generation of KG, making* ω*-amidase a valid therapeutic target.* This emphasizes the urgency for the development of ω-amidase inhibitors and their utilization either alone, or in combination with GLS inhibitors. It has been suggested that GTK inhibitors, either alone or in combination with a GLS1 inhibitor, may be alternative and effective anti-cancer agents (57, 59). While we agree with this premise, we propose that a potent, selective inhibitor of ω-amidase may be even more useful as an anti-cancer agent. Unfortunately, no such inhibitor currently exists. When Duffy et al. infused KGM into the CSF of rats, only minor neurological symptoms were noted, and only at relatively enormous concentrations (49). Thus, it is possible that inhibition of ω-amidase and accumulation of KGM may have only minimal (patho) physiological effects on normal tissues, whereas removal of a source of anaplerotic KG may be deleterious to tumors. *We strongly urge cancer researchers to consider the importance of the GT*ω*A pathway in cancer cell survival and prioritize the development of an* ω*-amidase inhibitor as a new weapon in the fight against cancer.*

## Author contributions

AJLC wrote the first draft. All authors contributed to the work quoted and approved the final draft.

## Footnotes

1
https://www.proteinatlas.org
